# COVID-19 Vaccine Effectiveness: A Review of the First 6 Months of COVID-19 Vaccine Availability (1 January–30 June 2021)

**DOI:** 10.3390/vaccines10030393

**Published:** 2022-03-03

**Authors:** Sarah M. Hatcher, Stacy M. Endres-Dighe, Frederick J. Angulo, Amit Srivastava, Jennifer L. Nguyen, Farid Khan, Catherine Martin, David L. Swerdlow, John M. McLaughlin, Nneka Ubaka-Blackmore, Linda Morris Brown

**Affiliations:** 1 RTI International, Research Triangle Park, NC 27709, USA; shatcher@rti.org (S.M.H.); sendres@rti.org (S.M.E.-D.); ncubaka@rti.org (N.U.-B.); lindabrown@rti.org (L.M.B.); 2 Pfizer, Inc., New York, NY 10017, USA; amit.k.srivastava@pfizer.com (A.S.); jennifer.nguyen2@pfizer.com (J.L.N.); farid.khan2@pfizer.com (F.K.); catherine.martin@pfizer.com (C.M.); dswerdlow@hsph.harvard.edu (D.L.S.); john.mclaughlin@pfizer.com (J.M.M.)

**Keywords:** COVID-19 vaccines, vaccine effectiveness, observational studies, review literature, SARS-CoV-2, BNT162b2 vaccine, mRNA-1273 vaccine, ChAdOx1 nCoV-19, Ad26.COV2.S

## Abstract

Observational studies are needed to demonstrate real-world vaccine effectiveness (VE) against severe acute respiratory syndrome coronavirus 2 (SARS-CoV-2) outcomes. Our objective was to conduct a review of published SARS-CoV-2 VE articles, supplemented by preprints, during the first 6 months of COVID-19 vaccine availability. This review compares the effectiveness of completing the primary COVID-19 vaccination series against multiple SARS-CoV-2 disease presentations and disease severity outcomes in three population groups (general population, frontline workers, and older adults). Four hundred and seventy-one published articles and 47 preprints were identified. After title and abstract screening and full article review, 50 studies (28 published articles, 22 preprints) were included. VE results were reported for five COVID-19 vaccines and four combinations of COVID-19 vaccines. VE results for BNT162b2 were reported in 70.6% of all studies. Seventeen studies reported variant specific VE estimates; Alpha was the most common. This comprehensive review demonstrates that COVID-19 vaccination is an important tool for preventing COVID-19 morbidity and mortality among fully vaccinated persons aged 16 years and older and serves as an important baseline from which to follow future trends in COVID-19 evolution and effectiveness of new and updated vaccines.

## 1. Introduction

The coronavirus disease 2019 (COVID-19) pandemic has caused significant morbidity, mortality, and economic loss globally. As a result, scientists around the world have been working tirelessly to develop, produce, and test COVID-19 vaccines that limit the spread of SARS-CoV-2 and prevent the adverse health effects of SARS-CoV-2 infection. Clinical trials have shown COVID-19 vaccines to be safe and immunogenic, with an efficacy against symptomatic infection in randomized controlled trials (RCTs) ranging from 95.0% and 94.1% for the messenger RNA (mRNA) vaccines BNT162b2 (Pfizer-BioNTech) [1] and mRNA-1273 (Moderna) [2], respectively, to 50.7% for the inactivated whole-virion vaccine CoronaVac (Sinovac) [3]. Other vaccines included in this review had intermediate efficacies of 77.8%, 67.1%, and 66.9%, for Covaxin^®^ (Bharat Biotech) [4], ChAdOx1 (AstraZeneca) [5], and Ad26.COV2.S (Janssen/Johnson & Johnson) [6], respectively. The first vaccine authorized and used in the United States (US) was BNT162b2; first doses were administered on 14 December 2020, and the first individuals completed the two-dose primary vaccination series in January 2021. Since vaccine trials, including the above-mentioned RCTs, are conducted in controlled settings with healthy individuals or those with stable medical conditions [1,2,3,4,5,6,7,8,9], observational studies are needed to demonstrate real-world vaccine effectiveness (VE) against all severe acute respiratory syndrome coronavirus 2 (SARS-CoV-2) outcomes, including asymptomatic infections and severe outcomes including hospitalizations and deaths, in field settings across the globe. It is also important to determine VE in subsets of the population who may be at higher risk of being infected with SARS-CoV-2 (e.g., frontline and healthcare workers) or having more severe outcomes (e.g., older adults and persons with underlying illnesses). Finally, it is important to monitor VE over time to assess changes in effectiveness, which may occur following waning immunity or the dissemination of SARS-CoV-2 variants that are associated with increased transmissibility or more severe illness.

We conducted a review of published (i.e., peer-reviewed) SARS-CoV-2 VE articles, supplemented by preprints posted on preprint servers and reports published on websites of public health agencies during the first 6 months of COVID-19 vaccine availability. While other VE reviews have been published [10,11,12,13,14], our review is unique in that we (1) provided VE results for the first 6 months of global vaccine use and for only fully vaccinated participants, (2) examined VE for three population groups separately, and (3) plotted VE results to allow for direct comparison across disease presentation and disease severity categories by vaccine and by days after full vaccination.

The objective of our review is to compare the effectiveness of completing the primary COVID-19 vaccine series (i.e., “fully vaccinated,” as defined during the period of this review) against multiple SARS-CoV-2 outcomes (i.e., infection, asymptomatic infection, symptomatic infection, hospitalization, severe disease, intensive care unit [ICU] admission, death) by vaccine product, study population, number of days after full vaccination, and variant. VE information assists physicians and public health officials with identifying which vaccines are most effective for which population subgroup and with monitoring trends to inform the need for subsequent vaccine doses.

## 2. Materials and Methods

A literature search was conducted in PubMed to identify articles published between 1 January and 30 June 2021, written in English, and describing observational studies that assessed VE against SARS-CoV-2 outcomes in real-world settings. This 6-month period was chosen to focus our review on VE among fully vaccinated persons aged 16 years and older without having to factor in the influence waning immunity or subsequent vaccine doses. In addition, this early time period provides an important baseline from which to follow future trends in COVID-19 evolution and effectiveness of new or updated vaccines.

**Figure 1:**
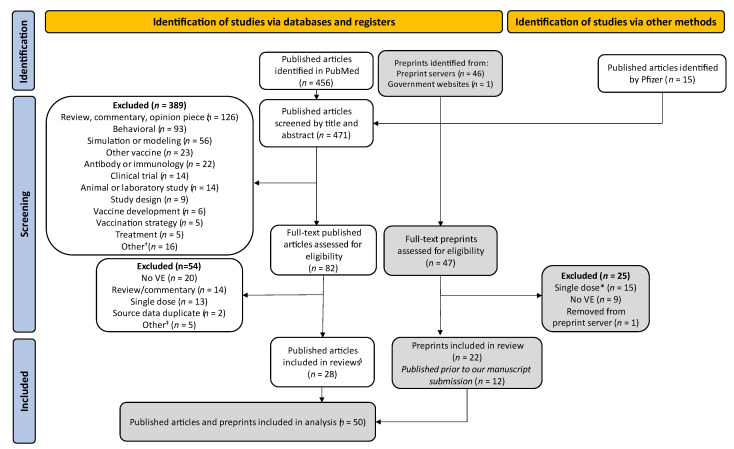
PRISMA Flow Chart. VE = vaccine effectiveness. ^†^ Economic or cost-effectiveness (*n* = 4); vaccine side effects (*n* = 4); case report or series (*n* = 3); surveillance study (*n* = 2); nutrition (*n* = 1); risk-benefit analysis (*n* = 1); symptoms (*n* = 1). ^‡^ Image or audio clip with no article (*n* = 2); news article (*n* = 2); author reply (*n* = 1). ^§^ Five identified by through sources other than PubMed. * One study did not distinguish between one- and two-dose VE.

The literature search terms are described in the Supplementary Methods. Separately, Pfizer investigators searched the medRxiv and bioRxiv COVID-19 SARS-CoV-2 preprint server and the SSRN preprint server daily for preprints of articles related to COVID-19 VE with the term “BNT162b2” or “effectiveness” in the title to identify preprints describing COVID-19 VE studies. Following a cursory review for appropriateness, preprint servers post scientific articles that have not yet been peer reviewed; such servers have been a vital mechanism for timely dissemination of scientific results during the rapidly evolving SARS-CoV-2 pandemic. Pfizer also monitored media reports and websites of national public health agencies daily to identify both published articles and preprints. These included reports from government agencies (e.g., Public Health England) that included COVID-19 VE information; for the purposes of this review, such reports are also considered as preprints. Published articles that were identified by Pfizer’s daily monitoring of media reports and websites of national public health agencies but were not identified through the PubMed search are referred to as “Published articles identified by Pfizer.” Published articles and preprints identified by the PubMed search and by Pfizer were included in the title and abstract screening and full article review process described below and summarized in Figure 1. Although we performed a comprehensive search of available literature as a part of our methods, we did not conduct a quality assessment of published articles and preprints. Thus, our review should not be considered a systematic literature review.

Published articles and preprints eligible for inclusion were observational studies that reported the effectiveness of any COVID-19 vaccine for fully vaccinated persons. The primary series for Ad26.COV2.S is one dose; all other vaccines are two doses. Two investigators (L.M.B. and S.M.H. or S.M.E.-D.) independently screened the titles and abstracts of all published articles, where available, to identify studies for a full article review. Published articles with no abstract or those with titles and abstracts that did not provide sufficient context to exclude them at the abstract review stage were included in the full article review. All preprints were included in the full article review. In the case of a disagreement, a third investigator (S.M.H. or S.M.E.-D.) reviewed the title and abstract to make a final determination about including or excluding the article.

One investigator (S.M.H. or S.M.E.-D.) reviewed full published articles and preprints for inclusion. Published articles and preprints were included if they presented VE or a measure from which VE could be directly calculated (i.e., incidence rate ratio [IRR], hazard ratio [HR], odds ratio [OR]). For published articles or preprints that provided an IRR, HR, or OR, VE was calculated using the formula (1-IRR/HR/OR) × 100. S.M.H., or S.M.E.-D. abstracted relevant data from articles and preprints selected for inclusion. Final abstracted data were reviewed by L.M.B. and S.M.H.

Among the 82 published articles assessed for eligibility, 20 were excluded because they did not present VE or a measure from which VE could be directly calculated, 14 were excluded because they were a review or commentary, 13 were excluded because they only presented VE for a single vaccine dose for vaccines with two-dose regimens, 2 were excluded because they contained data that were updated in a more recently published article (source data duplicate), and 5 were excluded for another reason detailed in Figure 1. Among the 47 preprints assessed for eligibility, 15 were excluded because they only presented VE for a single vaccine dose for vaccines with two-dose regimens, 9 were excluded because they did not present VE or a measure from which VE could be directly calculated, and 1 was excluded because it was removed from the preprint server prior to submission of this manuscript.

**Figure 2:**
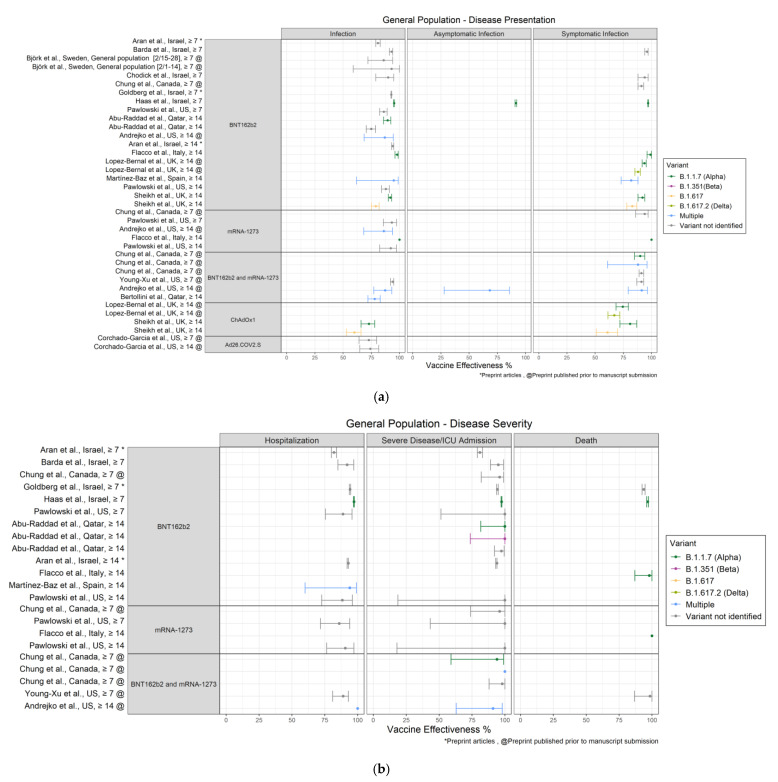
(**a**) Forest plot of VE estimates and 95% CIs against infection, asymptomatic infection, and symptomatic infection among the general population aged ≥16 years. (**b**) Forest plot of VE estimates and 95% CIs against hospitalization, severe disease or ICU admission, and death among the general population aged ≥16 years.

**Figure 3:**
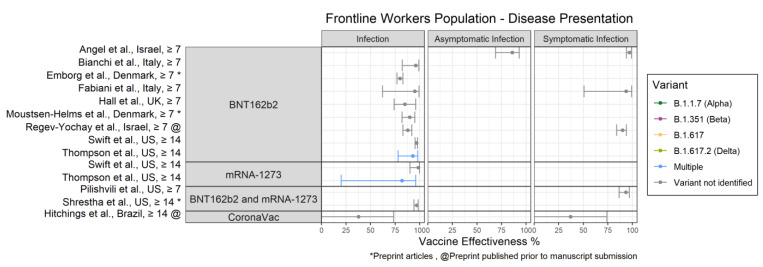
Forest plot of VE estimates and 95% CIs against infection, asymptomatic infection, and symptomatic infection among adult frontline workers.

**Figure 4:**
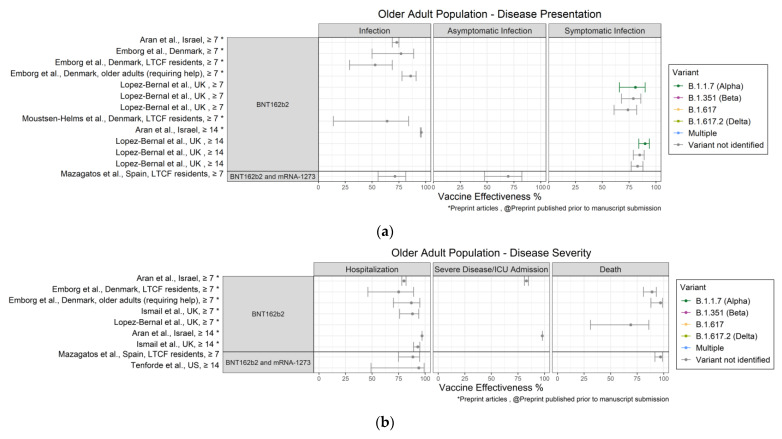
(**a**) Forest plot of VE estimates and 95% CIs against infection, asymptomatic infection, and symptomatic infection among older adults aged ≥65 years. (**b**) Forest plot of VE estimates and 95% CIs against hospitalization, severe disease or ICU admission, and death among older adults aged ≥65 years.

Abstracted information included country, study design, study period, study population, number of participants, participant age in years (mean, median, or category), number of participants vaccinated and unvaccinated, vaccine, number of days after being fully vaccinated, identified or circulating variant, and VE and 95% confidence intervals (CIs) by SARS-CoV-2 outcome. VE and 95% CIs were rounded to the nearest whole number. For published articles or preprints that provided VE for >1 vaccine and reported both combined and individual VE estimates, we reported only the individual results unless the combined VE estimates included additional stratification (e.g., by variant, disease presentation, disease severity) not provided for the individual VE estimates. A variant was considered “identified” if the study authors performed laboratory testing to identify the variant detected from each infected person, or a sample of infected persons, that contributed to the VE estimate. A variant was considered “circulating” if the study presented background information or other evidence of the dominant strain(s) circulating in the population during the study period but did not perform laboratory testing to identify variant(s) detected from infected persons. This detailed information is presented in Table 1 for each study. To compare VE results between populations with distinct disease or exposure risks, study populations were classified into three broad categories: general population aged ≥16 years, adult frontline workers, and older adults aged ≥65 years. One study [15] included in the older adults’ category persons aged >60 years. The link between the detailed study populations presented in Table 1 and the broad categories used in Table 2 and Figure 2, Figure 3 and Figure 4 is provided in Supplementary Table S1. To compare VE results by time after full vaccination, days after full vaccine dose were grouped into two categories: ≥7 days and ≥14 days (Figure 2, Figure 3 and Figure 4). For completeness, estimations of VE at <7 days are provided for two-dose regimens in Table 1.

Vaccine effectiveness estimates and 95% CIs were abstracted for both SARS-CoV-2 outcomes (infection, asymptomatic infection, or symptomatic infection) and disease severity outcomes (hospitalization, severe disease, ICU admission, or death). Adjusted VE results are presented unless otherwise specified. Studies are categorized and presented separately based on their source: “published articles” or “preprints.” For preprints that were published before manuscript submission, results were updated to reflect the published version of the article.

## 3. Results

Four hundred and seventy-one published articles and 47 preprints were identified. After title and abstract screening and full article review, 50 studies were included in this review, of which 28 were published articles and 22 were preprints (Figure 1). Of the 22 preprints, 12 were published prior to submission of this review. There was a change in the VE estimates between the preprint and published article for three articles [48,50,57] due to increases in participant numbers.

Characteristics of abstracted published articles and preprints included in the review are described in Table 2. Most studies were conducted in the US (26.0%), United Kingdom (UK) (22.0%), or Israel (16.0%). Adult frontline workers (44.0%) and the general population aged ≥16 years (42.0%) were the most common study populations, followed by older adults aged ≥65 years (22.0%). Overall, VE of five COVID-19 vaccines and four combinations of vaccines were reported, with BNT162b2 reported in 72.0% of all studies. Most studies estimated VE ≥7 days (56.0%) or ≥14 days (44.0%) after full vaccination. Seventeen studies (34.0%) reported VE estimates for specific identified variants (10 for single variants, 6 for multiple variants combined, and 1 for both single and multiple variants). Alpha (B.1.1.7) was the most common identified variant reported (58.8%), followed by Delta (B.1.617.2) (11.8%); SARS-CoV-2 variants B.1.351, B.1.617, and R.1 were each reported by one study. Nine studies (18.0%) reported circulating variant specific VE estimates (5 for single variants, 4 for multiple variants combined). Alpha (B.1.1.7) was also the most common circulating variant reported (55.6%); one study reported VE when the P.1 variant was circulating. In all studies, the most common SARS-CoV-2 outcomes reported were infection (74.0%), symptomatic infection (44.0%), and asymptomatic infection (18.0%). The most common disease severity outcomes reported were hospitalization (32.0%), death (22.0%), and ICU admission or severe disease (20.0%).

Characteristics of all 50 published articles and preprints that assessed VE are provided in Table 1. Results are presented in alphabetic order by study author [15,16,17,18,19,20,21,22,23,24,25,26,27,28,29,30,31,32,33,34,35,36,37,38,39,40,41,42,43,44,45,46,47,48,49,50,51,52,53,54,55,56,57,58,59,60,61,62,63,64,65,66] under the headings Published Articles and Preprints. Vaccine effectiveness estimates for identified and circulating variants are also provided. In Figure 2, Figure 3 and Figure 4, VE estimates are presented separately by population group for disease presentation and disease severity, and are stratified by vaccine (BNT162b2, mRNA-1273, BNT162b2 and mRNA-1273, ChadOx1, Ad26.COV2.S, CoronaVac) and days after final dose (≥7 and ≥14). Where available, variant specific VE results are shown for variants of concern (VOCs) [67]: Alpha, Beta (B.1.351), Delta (B.1.617.2), and other recorded variants, including B.1.617, R.1, and multiple variants.

### 3.1. General Population Aged ≥16 Years

Vaccine effectiveness results for the general population aged ≥16 years by disease presentation are presented in Figure 2a.

#### 3.1.1. Overall Results by Vaccine

For BNT162b2, VE against infection ranged from 75% (95% CI: 70.5%–78.9%) [16] to 98% (95% CI: 96–99%) [25]. Aran et al. [15] reported a VE of 81% (95% CI: 79–83%) and 94% (95% CI: 93–95%) for ≥7 days and ≥14 days since full vaccination, respectively. VE against symptomatic infection ranged from 82% (95% CI: 73–88%) [31] to 99% (95% CI: 96–100%) [25], and VE against asymptomatic infection was 92% (95% CI: 91–92%) [26]. For mRNA-1273, VE against infection ranged from 86% (95% CI: 68–94%) [46] to 100% (95% CI not specified) [25], and VE against symptomatic infection was 94% (95% CI: 86–97%) [49] and 100% (95% CI not specified) [25]. For studies that presented combined results for both mRNA vaccines, VE against infection ranged from 78% (95% CI: 72–83%) [20] to 94% (95% CI: 92–95%) [66] and VE against symptomatic infection ranged from 88% (95% CI: 61–96%) to 93% (95% CI: 87–96%) [49]. Andrejko et al. [46] reported a VE against asymptomatic infection of 68% (95% CI: 28–86%). Corchado-Garcia et al. [50] reported that Ad26.COV2.S VE against infection was 73% (95% CI: 64–80%) >7 days after vaccination and 74.2% (95% CI: 65–82%) ≥14 days after vaccination.

#### 3.1.2. Identified Variant-Specific Results

For the Alpha variant, VE against infection ranged from 73% (95% CI: 66–78%) for the ChAdOx1 vaccine [37] to 100% (95% CI not specified) for the mRNA-1273 vaccine [25]. VE against symptomatic infection ranged from 75% (95% CI: 68–79%) for the ChAdOx1 vaccine [57] to 100% (95% CI not specified) for the mRNA-1273 vaccine [25]. Haas et al. [26] reported that BNT162b2 VE against asymptomatic infection with the Alpha variant was 92% (95% CI: 91–92%). For the Beta variant, VE against infection for BNT162b was 75% (90% CI: 71–79%) [16]. For the Delta variant, VE against symptomatic infection ranged from 67% (95% CI: 61–72%) for the ChAdOx1 vaccine to 88% (95% CI: 85–90%) for the BNT162b2 vaccine [57]. Sheikh et al. [37] reported that for the B.1.617 variant, the ChAdOx1 VE was 60% (95% CI: 53–66%) against infection and 61% (95% CI: 51–70%) against symptomatic infection; BNT162b2 VE was 79% (95% CI: 75–83%) against infection and 83% (95% CI: 78–87%) against symptomatic infection.

Figure 2b presents the VE results for the general population aged ≥16 years by disease severity.

#### 3.1.3. Overall Results by Vaccine

For BNT162b2, VE against hospitalization ranged from 82% (95% CI: 80–84%) [15] to 97% (95% CI: 97–98%) [26]. VE against severe disease or ICU admission ranged from 81% (95% CI: 79–83%) [15] to 100% [16,33]; the majority of results for VE against severe disease or ICU admission were 94% or greater [15,16,18,26,33,49,53]. Results by Aran et al. [15] showed noteworthy differences in VE at >7 days (hospitalization: 82% [95% CI: 80–84%]; severe disease or ICU admission: 81% [95% CI: 79–83%]) compared with >14 days (hospitalization: 93% [95% CI: 92–93%]; severe disease or ICU admission: 94% [95% CI: 93–94%]) since full vaccination. In the three studies that reported VE against death, VE ranged from 94% (95% CI: 93–95%) [53] to 98% (95% CI: 87–100%) [25]. For mRNA-1273, VE estimates against hospitalization, severe disease or ICU admission, and death were all 86% or greater [25,33,49]. For combined mRNA vaccine, VE against hospitalization ranged from 89% (95% CI: 81–93%) [66] to 100% (95% CI not specified) [46] and VE against severe disease or ICU admission ranged from 90% (95% CI: 61–98%) to 100% (95% CI not specified) [49]. Young-Xu et al. [66] reported a VE against death of 99% (95% CI: 87–100%).

#### 3.1.4. Identified Variant-Specific Results

For the Alpha variant, the VE against hospitalization was 97% (95% CI: 97–98%) for the BNT162b2 vaccine [26], VE against severe disease or ICU admission ranged from 94% (95% CI: 59–99%) for the combined mRNA vaccines [49] to 100% (95% CI: 82–100%) for BNT162b2 vaccine [16], and VE against death ranged from 96.7% (95% CI: 96–97.3%) for the BNT162b2 vaccine [26] to 100% (95% CI not specified) for the mRNA-1273 vaccine [25]. For the Beta variant, VE against severe disease or ICU admission was 100% (95% CI: 74–100%) for BNT162b2 [16].

### 3.2. Frontline Workers

Vaccine effectiveness results for frontline workers by disease presentation are shown in Figure 3. The only VE estimates for disease severity were reported for the combined ChAdOx1 and Covaxin vaccines [44] (see Table 1).

#### 3.2.1. Overall Results by Vaccine

For BNT162b2, VE against infection ranged from 80% (95% CI: 77–83%) [52] to 97% (95% CI: 95–98%) [38]. VE against symptomatic infection was ≥90% in all three studies that reported it [17,24,62]. Angel et al. [17] reported that VE against asymptomatic infection was 86% (95% CI: 69–93%). For mRNA-1273, VE against infection was reported in two studies and ranged from 82% (95% CI: 20–96%) [42] to 99% (95% CI: 90–100%) [38]. For combined mRNA vaccine, VE against infection was 97% (95% CI: 94–99%) [64] and VE against symptomatic infection was 94% (95% CI: 87–97%) [34]. For CoronaVac, VE against infection was 38% (95% CI: −46% to 74%) and VE against symptomatic infection was 37% (95% CI: −53% to 74%) [55].

#### 3.2.2. Identified Variant-Specific Results

For the R.1. variant, the crude BNT162b2 VE against infection and symptomatic infection was 76% (95% CI: 33–91%) and 87% (95% CI: 46–97%), respectively [22].

### 3.3. Older Adults Aged ≥65 Years

Disease presentation VE estimates for older adults are illustrated in Figure 4a.

#### 3.3.1. Overall Results by Vaccine

For BNT162b2, VE against infection ranged from 53% (95% CI: 29–69%) [52] to 96% (95% CI: 95–96%) [15]; Aran et al. [15] reported that BNT162b2 VE against infection was 73% (95% CI: 69–75%) and 96% (95% CI: 95–96%) >7 days and >14 days since full vaccination, respectively. For combined mRNA vaccines, VE against infection was 71% (95% CI: 56–82%) and VE against asymptomatic infection was 70% (95% CI: 48–83%) [32].

#### 3.3.2. Identified Variant-Specific Results

For the Alpha variant, BNT162b2 VE against symptomatic infection was 81% (95% CI: 66–90%) and 90% (95% CI: 84–94%) ≥7 days and ≥14 days after full vaccination, respectively [30]. For the R.1 variant, BNT162b2 crude VE was 66% (95% CI: 41–81%) against infection and 86.5% (95% CI: 66–95%) against symptomatic infection [22].

Shown in Figure 4b and Table 1 are the VE severity estimates for older adults.

#### 3.3.3. Overall Results by Vaccine

For BNT162b2, VE against hospitalization ranged from 75% (95% CI: 46–89%) [52] to 97% (95% CI: 97–97%) [15]. Aran et al. [15] reported that VE against hospitalization was 80% (95% CI: 78–82%) and 97% (95% CI: 97–97%) >7 and >14 days since full vaccination, respectively. VE against severe disease or ICU admission was 83% (95% CI: 81–85%) and 98% (95% CI: 98–98%) >7 and >14 days since full vaccination, respectively [15]. VE against death ranged from 69% (95% CI: 31–86%) [58] to 97% (97% CI: 88–99%) [52]. For combined mRNA vaccines, VE against hospitalization was reported in two studies and ranged from 88% (95% CI: 75–95%) [32] to 94% (95% CI: 49–99%) [41]. VE against death was 97% (95% CI: 92–99%) [32].

#### 3.3.4. Identified Variant-Specific Results

For the R.1 variant, the BNT162b2 crude VE against hospitalization and death was 94% (95% CI: 74–99%) and 94% (95% CI: 45–99%), respectively [22] (Table 1).

## 4. Discussion

This review included 50 real-world studies encompassing both published peer-reviewed articles and preprints conducted among participants aged 16 years and older during the first 6 months of COVID-19 vaccine use worldwide. Including preprints in the review enabled the capture of timelier COVID-19 research in this rapidly evolving field. Of the 23 preprints initially identified, 12 were published prior to submission of this article, and in only a few instances were minor updates to the VE results required. While other VE reviews have been published [10,11,12,13,14], our review was unique in that we (1) provided VE results for the first 6 months of global vaccine use and for only fully vaccinated participants, (2) examined VE for three population groups separately, and (3) plotted VE results to allow for direct comparison across disease presentation and disease severity categories by vaccine and by days after full vaccination. For a global population that was immunologically naïve to SARS-CoV-2, our focus on VE among fully vaccinated persons aged 16 years and older is valuable because it provides a baseline to compare the effectiveness of full vaccination in various populations around the world without having to factor in the influence of waning immunity [68,69], novel variants [70], or subsequent vaccine doses. Future reviews of VE over longer time frames and within the context of VOC and subsequent vaccine doses will help address these important topics and help guide public health recommendations.

The real-world studies in our review indicate that among fully vaccinated persons, the mRNA vaccines BNT162b2 and mRNA-1273 were highly effective, particularly in preventing severe outcomes of SARS-CoV-2 infection. For example, among the general population aged ≥16 years BNT162b2 VE estimates were ≥82%, ≥81%, and ≥94% against hospitalization, severe disease or ICU admission, and death, respectively, and mRNA-1273 VE estimates were ≥86% against all disease severity categories. Among older adults, BNT162b2 VE was ≥75% and ≥69% against hospitalization and death, respectively, and combined mRNA vaccines VE was ≥88% and ≥97% against hospitalization and death, respectively. Although most VE estimates were similar for the two time periods ≥7 and ≥14 days after full vaccination, a large study by Aran et al. [15] noted significantly higher VE estimates for the BNT162b2 vaccine in the general population aged ≥16 years and in adults aged ≥65 years for infection, hospitalization, and severe disease for vaccination >14 days compared with 7–13 days after full vaccination. A possible reason why these time period differences were observed only by Aran et al. [15] is their use of distinct time periods (i.e., 7–13 days and ≥14 days rather than ≥7 days and ≥14 days).

ChAdOx1 VE values against SARS-CoV-2 infection (≥60%) were generally lower than those for the mRNA vaccines; however, ChAdOx1 provided similarly strong protection against severe disease compared with the mRNA vaccines, including among frontline workers. In contrast, CoronaVac effectiveness against infection (38%) and symptomatic infection (37%) were substantially lower than mRNA vaccines and ChAdOx1. This lower VE is not unexpected given the relatively lower efficacy reported in CoronaVac RCTs in Turkey and Brazil [3,7]. Our review included only one study that reported VE for Ad26.COV2.S. This is likely because the US Food and Drug Administration and World Health Organization did not authorize Ad26.COV2.S for emergency use until 27 February 2021 [71], and 11 March 2021 [72], respectively.

Although we collected variant-specific information, our review predominantly included studies from Israel, the UK, and the US during the time period when the Alpha variant was the only VOC that broadly disseminated in these countries. Alpha was first identified in the UK in September 2020 and was the predominant variant globally between January and May 2021 [57,65]. Alpha became the dominant variant in Israel in December 2020 and in the US in April 2021. Although Beta, Gamma, and Delta variants were detected in South Africa, Brazil, and India during our study period, their prevalence in Israel, the UK, and the US during our study period was low [57,65].

This review was subject to several limitations. We did not perform a rigorous evaluation of study quality; as a result, some errors in study design or analysis may not have been identified. We did not attempt to perform meta-analyses due to the heterogeneity in study design, study analysis methods, study populations, circulating variants, time of VE assessment, and other variables that limit VE comparison among studies [73]. For example, there was variation in the timetable that the vaccine was available in each subgroup (i.e., frontline workers were offered the vaccine ahead of the general population) and the type of vaccine available in each country (e.g., ChAdOx1 vaccine is not authorized for use in the US). Information about the effectiveness of vaccination among previously infected persons was not abstracted, and we did not evaluate whether studies included previously infected persons in their vaccinated or unvaccinated groups. Some studies estimated VE by pooled analysis of two or more vaccines, and the proportion of the study population receiving each vaccine was often unevenly distributed; in these cases, pooled estimates might underestimate or overestimate the VE for one or more vaccines. Although we categorized study populations based on exposure or disease severity risk, heterogeneity with respect to exposure and disease severity risk within our population groups exists. We were unable to assess the impact of waning immunity on VE because our study focuses on the first 6 months of vaccine use; thus, few study participants had been fully vaccinated for >4 months. Finally, 10 studies included in this review have not yet been published and thus have not been certified by peer review.

Despite these limitations, this review of the effectiveness of COVID-19 vaccines in the first 6 months of vaccine use demonstrates that COVID-19 vaccination is an important tool for preventing COVID-19 morbidity and mortality. We found that mRNA vaccines are highly effective at preventing severe outcomes of SARS-CoV-2 infection, including among vulnerable populations such as older adults. As we limited our review to studies that reported VE among fully vaccinated persons aged 16 years and older, it serves as an important baseline from which to follow future trends in COVID-19 evolution and effectiveness of new or updated vaccines. To better understand the broader vaccine landscape, future reviews should include observational studies from a wider range of countries of new or updated vaccines as they become more widely available, and of adolescents and children. They should also include studies that evaluate the impact of VOC, comorbidities, waning immunity, and subsequent vaccine doses on VE.

## 5. Conclusions

This comprehensive review of 50 real-world studies conducted during the first 6 months of COVID-19 vaccine use worldwide demonstrates that COVID-19 vaccination, particularly with the mRNA vaccines, is an important tool for preventing COVID-19 morbidity and mortality among fully vaccinated persons aged 16 years and older, including among vulnerable populations. This review also serves as an important baseline from which to follow future trends in COVID-19 evolution and effectiveness of new and updated vaccines.

## Figures and Tables

**Table 1:** Characteristics of published articles (*n* = 28) and preprints (*n* = 22) that assessed VE of COVID-19 vaccines against SARS-CoV-2 outcomes, 1 January–30 June 2021.

Study (Country)	Study Design	Study Period	Study Population (Age in Years)	Participant Number	Participant Age in Years	Participants Vaccinated (Unvaccinated)	Vaccine Received	Days after Full Vaccine Dose	Variant Identified (Bold) or *Circulating (Italics)*	VE	
										Outcome	Adjusted VE, % (95% CI)
**Published articles**											
Abu-Raddad et al. [16] (Qatar)	Test-negative case–control	1 Feb 2021–31 Mar 2021	General population (not specified)	75,318	32.5 (median)	515 (32,293)	BNT162b2	>14	**B.1.1.7**	Infection	90 (86–92)
						75 (727)	BNT162b2	>14	**B.1.1.7**	Severe disease	100 (82–100)
						877 (38,273)	BNT162b2	>14	**B.1.351**	Infection	75 (71–79)
						14 (586)	BNT162b2	>14	**B.1.351**	Severe disease	100 (74–100)
						112 (3278)	BNT162b2	>14	*B.1.1.7, B.1.351*	Severe disease	97 (92–100)
	Retrospective cohort	1 Feb 2021–31 Mar 2021	General population (not specified)	213,758	Vaccinated 54 (median); unvaccinated37 (median)	51,324 (162,434)	BNT162b2	>14	**B.1.1.7**	Infection	87 * (82–91)
									**B.1.351**	Infection	72 * (66–77)
									*B.1.1.7, B.1.351*	Infection	69 * (63–74)
Angel et al. [17] (Israel)	Retrospective cohort	20 Dec 2020–25 Feb 2021	HCWs (18+)	6274	44.3 (mean)	5517 (757)	BNT162b2	>7	*B.1.1.7*	Asymptomatic	86 (69–93)
										Symptomatic	97 (94–99)
Barda et al. [18] (Israel) [includes data from: Dagan et al. [19], (Israel)]	Retrospective cohort	20 Dec 2020–14 Feb 2021	HS members (16+)	310,696	Not provided	155,348 (155,348)	BNT162b2	7–28	*B.1.1.7*	Infection	93 (91–94)
										Symptomatic	96 (94–97)
										Hospitalization	92 (85–97)
										Severe disease	95 (89–99)
Bertollini et al. [20] (Qatar) ++	Cross-sectional	18 Feb 2021–26 Apr 2021	Airline passengers (not provided)	20,184	33 (median)	10,092 (10,092)	BNT162b2, mRNA-1273	>14	**B.1.1.7, B.1.351, B.1.617, “wildtype”** **strains**	Infection	78 (72–83)
Bianchi et al. [21] (Italy)	Prospective cohort	24 Jan 2021–31 Mar 2021	HCWs (18+)	2034	44.4 (mean)	1607 (427)	BNT162b2	>7	NA	Infection	96 82–99
Cavanaugh et al. [22] (US)	Outbreak investigation	1 Mar 2021–17 Mar 2021	SNF residents (not provided)	79	Not provided	71 (8)	BNT162b2	>14	**R.1**	Infection	66 * (41–81)
										Symptomatic	87 * (66–95)
										Hospitalization	94 * (74–99)
										Death	94 * (45–99)
		1 Mar 2021–28 Mar 2021	SNF HCWs (not provided)	108	Not provided	54 (54)	BNT162b2	>14	**R.1**	Infection	76 * (33–91)
										Symptomatic	87 * (46–97)
Chodick et al. [23] (Israel)	Retrospective cohort	20 Dec 2020–3 Mar 2021	HS members (16+)	2,051,051	47.7 (mean)	Ref period: 1,178,597 (protection period 872,454)	BNT162b2	>7	NA	Infection	90 (79–95)
										Symptomatic	94 (88–97)
Fabiani et al. [24] (Italy)	Retrospective cohort	27 Dec 2020–24 Mar 2021	HCWs (not provided)	6276	47.1 (mean)	5186 (1090)	BNT162b2	>7	NA	Infection	95 (62–99)
										Symptomatic	94 (51–99)
Flacco et al. [25] (Italy)	Retrospective cohort	17 Jan 2021–21 May 2021	General population (18+)	206,860	53.2 (mean)	30,817 (174,023)	BNT162b2	>14	**B.1.1.7**	Infection	98 (96–99)
										Symptomatic	99 (96–100)
										Death	98 (87–100)
						2020 (174,023)	mRNA-1273	>14	**B.1.1.7**	Infection	100
										Symptomatic	100
										Death	100
Haas et al. [26] (Israel)	Retrospective cohort	24 Jan 2021–3 Apr 2021	General population (16+)	6,538,911	Not provided	4,714,932 (1,823,979)	BNT162b2	>7	**B.1.1.7**	Infection	95 (95–96)
										Asymptomatic	92 (91–92)
										Symptomatic	97.0 (96.7–97.2)
										Hospitalization	97 (97–98)
										ICU	98 (97–98)
										Death	97 (96–97)
Hall et al. [27] (UK)	Prospective cohort	7 Dec 2020–5 Feb 2021	HCWs (18+)	23,324	46.1 (median)	Cohort+: 8203/cohort-: 15,121	BNT162b2	7	*B.1.1.7*	Infection	85 (74–96)
Khan et al. [28] (US)	Retrospective cohort	18 Dec 2020–20 Apr 2021	Veterans with IBD/ immunosuppressed (18+)	13,629	Not provided	6253 (7376)	BNT162b2, mRNA-1273	>7	NA	Infection	80
										Death	87
										Severe disease	70
Knobel et al. [29] (Spain)	Prospective cohort	1 Dec 2020–20 Apr 2021	HCWs (not specified)	2462	38.9 (mean)	2148 ** (314)	BNT162b2, mRNA-1273	>7	NA	Asymptomatic	91 *
Lopez Bernal et al. [30] (UK)	Test-negative case–control	8 Dec 2020–18 Jan 2021	Older adults (70+)	25,610	80+	675 (24,706)	BNT162b2	>14	*B.1.1.7*	Symptomatic	85 (79–89)
						229 (24,706)	BNT162b2	7–13			79 (68–86)
				127,656	70+	714 (126,697)	BNT162b2	>14	*B.1.1.7*	Symptomatic	83 (77–88)
						245 (126,697)	BNT162b2	7–13			74 (61–82)
				109,371	70+	411 (10,822)	BNT162b2	>14	**B.1.1.7**	Symptomatic	90 (84–94)
						138 (10,822)	BNT162b2	7–13			81 (66–90)
Martínez-Baz et al. [31] (Spain)	Prospective cohort	1 Jan 2021–30 Apr 2021	HS members (close contacts) (18+)	20,092	Not provided	512 (19,580)	BNT162b2, mRNA-1273, ChAdOx1	14	**B.1.1.7, B.1.177, P.1, B.1.351**	Infection	66 (57–74)
										Symptomatic	82 (74–88)
										Hospitalization	98 (87–100)
						491 (19,580)	BNT162b2	14	**B.1.1.7, B.1.177, P.1, B.1.351**	Infection	95 (62–99)
										Symptomatic	82 (73–88)
										Hospitalization	94 (60–99)
Mazagatos et al. [32] (Spain)	Case-coverage	27 Dec 2020–4 Apr 2021	LTCF residents (65+)	338,145	Not provided	300,133 (38,012)	BNT162b2, mRNA-1273	BNT16b2: >7; mRNA-1273: >14	NA	Infection	71 (56–82)
										Asymptomatic	70 (48–83)
										Hospitalization	88 (75–95)
										Death	97 (92–99)
Pawlowski et al. [33] (US)	Retrospective cohort	1 Dec 2020–20 Apr 2021	HS members (18+)	181,746	53.6 (mean)	33,963 (32,910)	BNT162b2	>14	NA	Infection	88 (84–91)
										Hospitalization	88 (73–96)
										ICU	100 (19–100)
						35,990 (35,011)	BNT162b2	>7	NA	Infection	86 (82–89)
										Hospitalization	89 (76–96)
										ICU	100 (51–100)
					62.6 (mean)	10,610 (10,318)	mRNA-1273	>14	NA	Infection	92 (82–97)
										Hospitalization	91 (77–97)
										ICU	100 (18–100)
						11,612 (11,332)	mRNA-1273	>7	NA	Infection	93 (86–97)
										Hospitalization	86 (72–94)
										ICU	100 (43.3–100)
Pilishvili et al. [34] (US)	Test-negative case–control	1 Jan 2021–30 Mar 2021	HCWs (19+)	845	37 (median)	203 (642)	BNT162b2, mRNA-1273	>7	NA	Symptomatic	94 (87–97)
Pritchard et al. [35] (UK)	Prospective cohort	1 Dec 2020–8 May 2021	General population (16+)	290,888	55 (median)	57,646 (192,224)	BNT162b2	>1	**B.1.1.7**	Infection	80 (74–84)
										Asymptomatic	58 (43–69)
										Symptomatic	95 (91–98)
						41,018 (192,224)	ChAdOx1	>1	**B.1.1.7**	Infection	79 (65–88)
										Asymptomatic	61 (27–79)
										Symptomatic	92 (78–97)
Sansone et al. [36] (Italy)	Surveillance study	25 Jan 2021–13 Apr 2021	HCWs (not provided)	8851	Not provided	6904 (1942)	BNT162b2	>7	**B.1.1.7,** **B.1.525**	Infection	61 * (9–83)
Sheikh et al. [37] (UK)	Test-negative cohort	1 Apr 2021–6 Jun 2021	General population (16+)	504,658	Not provided	53,575 (119,419)	BNT162b2	>14	**B.1.1.7**	Infection	92 (90–93)
						4360 (42,062)	BNT162b2	>14	**B.1.1.7**	Symptomatic	92 (88–94)
						53,679 (117,263)	BNT162b2	>14	**B.1.617**	Infection	79 (75–82)
						4401 (40,504)	BNT162b2	>14	**B.1.617**	Symptomatic	83 (78–87)
						32,588 (119,419)	ChAdOx1	>14	**B.1.1.7**	Infection	73 (66–78)
						1999 (42,062)	ChAdOx1	>14	**B.1.1.7**	Symptomatic	81 (72–87)
						32,719 (117,263)	ChAdOx1	>14	**B.1.617**	Infection	60 (53–66)
						2,089 (40,504)	ChAdOx1	>14	**B.1.617**	Symptomatic	61 (51–70)
Swift et al. [38] (US)	Retrospective cohort	1 Jan 2021–31 Mar 2021	HCWs (not provided)	69,093	41 (median)	41,741 (23,931)	BNT162b2	>14	NA	Infection	97 (95–98)
						3421 (23,931)	mRNA-1273	>14	NA	Infection	99 (90–100)
Tande et al. [39] (US) ++	Retrospective cohort	17 Dec 2020–8 Feb 2021	HS members (18+)	39,156 (46,034 screenings)	54.2 (mean)	Screenings: 707 (45,327)	BNT162b2, mRNA-1273	>0	NA	Asymptomatic	80 (56–91)
						Screening: 665 estimated(45,327)	BNT162b2	>0	NA	Asymptomatic	80 (56–91)
Tang et al. [40] (US) ++	Prospective cohort	17 Dec 2020–20 Mar 2021	HCWs (not provided)	4441	Not provided	2276 (2165)	BNT162b2	>7	NA	Infection	96 * (91–98)
										Asymptomatic	90 * (78–96)
										Symptomatic	100 *
Tenforde et al. [41] (US)	Test-negative case–control	1 Jan 2021–26 Mar 2021	Hospital patients (65+)	306	73 (median)	19 (287)	BNT162b2, mRNA-1273	14	NA	Hospitalization	94 (49–99)
Thompson et al. [42] (US) [includes data from: Thompson et al. [43] (US)]	Prospective cohort	14 Dec 2020–10 Apr 2021	HCWs, first responders, essential workers (18–85)	3482	Not provided	1800 (67% of 2686) (796)	BNT162b2	>14	**B.1.1.7, B.1.427, B.1.429, P.2**	Infection	93 (78–98)
						886 (33% of 2686) (796)	mRNA-1273	>14	**B.1.1.7, B.1.427, B.1.429, P.2**	Infection	82 (20–96)
Victor et al. [44] (India) ^++^	Prospective cohort	21 Feb 2021–19 May 2021	HCWs (not provided)	8689	Not provided	7080 (1609)	ChAdOx1, Covaxin	14	NA	Infection	65 * (61–68)
										Hospitalization	77 * (68–84)
										ICU	94 * (73–99)
										Severe disease	92 * (74–97)
Zacay et al. [45] (Israel)	Retrospective cohort	1 Jan 2021–11 Feb 2021	HS members (16+)	4841	Vaccinated 52 (mean); unvaccinated 36 (mean)	2941 (1900)	BNT162b2	>7	NA	Infection	89 * (82–94)
**Preprints**											
Andrejko et al. [46] (US) ^@^	Test-negative case–control	24 Feb 2021–29 Apr 2021	General population (18+)	873	Not provided	106 (767)	BNT162b2, mRNA-1273	>15	**B.1.1.7,** **B.1.427, B.1.429**	Infection	87 (77–93)
										Asymptomatic	68 (28–86)
										Symptomatic	91 (79–96)
										Hospitalization	100
										Severe disease	91 (63, 98)
							BNT162b2	>15	**B.1.1.7, B.1.427, B.1.429**	Infection	87 (69–95)
							mRNA-1273	>15	**B.1.1.7, B.1.427, B.1.429**	Infection	86 (68–94)
Aran et al. [15] (Israel)	Retrospective cohort	20 Dec 2020–9 Feb 2021	Older adults (60+)	4,671,315 **	Not provided	2,918,008 ** (1,753,307)	BNT162b2	>14	NA	Infection	96 (95–96)
										Hospitalization	97 (97–97)
										Severe disease	98 (98–98)
			General population (<60)			2,918,008 ** (1,753,307)	BNT162b2	>14	NA	Infection	94 (93–95)
										Hospitalization	93 (92–93)
										Severe disease	94 (93–94)
			Older adults (60+)			2,918,008 ** (1,753,307)	BNT162b2	7–13	NA	Infection	73 (69–75)
										Hospitalization	80 (78–82)
										Severe disease	83 (81–85)
			General population (<60)			2,918,008 ** (1,753,307)	BNT162b2	7–13	NA	Infection	81 (79–83)
										Hospitalization	82 (80–84)
										Severe disease	81 (79–83)
Björk et al. [47] (Sweden) ^@^	Prospective cohort	27 Dec 2020–28 Feb 2021	General population (18–64) (2/15–2/28)	805,741 **	Vaccinated 47 (median); unvaccinated 40 (median)	26,587 ** (779,154)	BNT162b2	>7	NA	Infection	86 (72–94)
			General population (18–64) (2/1–2/14)	805,741 **		26,587 ** (779,154)	BNT162b2	>7	NA	Infection	93 (59–100)
Cabezas et al. [48] (Spain) ^@^	Prospective cohort	27 Dec 2020–5 Mar 2021	LTCF residents	28,456 **	86 (mean)	26,987 ** (1469)	BNT162b2	>0	NA	Infection	91 (89–92)
										Hospitalization	95 (93–96)
										Death	97 (96–98)
			LTCF staff	26,170 **	44 (mean)	21,870 ** (4300)	BNT162b2	>0	NA	Infection	80 (76–83)
			HCWs	61,791 **	43 (mean)	55,790 ** (6001)	BNT162b2	>0	NA	Infection	87 (84–89)
Chung et al. [49] (Canada) ^@^	Test-negative case–control	14 Dec 2020–19 Apr 2021	General population (16+)	307,655	Not provided	4894 (302,761)	BNT162b2, mRNA-1273	>7	*B.1.1.7, B.1.351, P.1*	Symptomatic	91 (89–93)
										Severe disease	98 (88–100)
							BNT162b2, mRNA-1273	>7	**B.1.1.7**	Symptomatic	90 (85–94)
										Severe disease	94 (59–99)
							BNT162b2, mRNA-1273	>7	**B.1.351, P.1**	Symptomatic	88 (61–96)
										Severe disease	100
							BNT162b2, mRNA-1273	>7	**“Earlier variant”**	Symptomatic	93 (87–96)
										Severe disease	90 (61–98)
							BNT162b2	>7	*B.1.1.7, B.1.351, P.1*	Symptomatic	91 (88–93)
										Severe disease	96 (82–99)
							mRNA-1273	>7	*B.1.1.7, B.1.351, P.1*	Symptomatic	94 (86–97)
										Severe disease	96 (74–100)
Corchado-Garcia et al. [50] (US) ^@^	Comparative effectiveness	27 Feb 2021–22 Jul 2021	HS members (18+)	97,787	Vaccinated 52.4 (mean); unvaccinated 51.7 (mean)	(86,495)	Ad26.COV2.S	>15	*B.1.1.7, B.1.617.2*	Infection	74 (65–82)
						8834 (88,052)		>8		Infection	73 (64–80)
de Faria et al. [51] (Brazil)	Prospective cohort	23 Feb 2021–28 Mar 2021	Vaccinated HCWs and general population (not provided)	HCWs: 21,652 General: 11,069,605	Not provided	HCWs: 21,652 (NA) General: 437,438 (10,632,167)	CoronaVac	14	**B.1.1.7, P.1, other VOC**	Symptomatic	51 * (33–63)
Emborg et al. [52] (Denmark)	Retrospective cohort	27 Dec 2020–11 Apr 2021	LTCF residents, older adults, HCWs, severe risk individuals	790,762	Not provided	400,623 (390,139)	BNT162b2	>7	NA	Infection	82 (79–84)
										Hospitalization	93 (89–96)
										Death	94 (9–96)
			LTCF residents	43,418	Vaccinated 84 (median); unvaccinated not provided	40,061 (3357)	BNT162b2	>7	NA	Infection	53 (29–69)
										Hospitalization	75 (46–89)
										Death	89 (81–93)
			Older adults requiring help (65+)	56,436	Vaccinated: 83 (median); unvaccinated not provided	45,942 (10,494)	BNT162b2	>7	NA	Infection	86 (78–91)
										Hospitalization	87 (70–95)
										Death	97 (88–99)
			Older adults (85+)	132,172	Vaccinated: 86 (median); unvaccinated not provided	112,824 (19,348)	BNT162b2	>7	NA	Infection	77 (50–89)
			HCWs	381,345	Vaccinated: 49 (median); unvaccinated not provided	75,497 (305,848)	BNT162b2	>7	NA	Infection	80 (77–83)
			Severe risk individuals	177,391	Vaccinated: 68 (median); unvaccinated not provided	126,299 (51,092)	BNT162b2	>7	NA	Infection	71 (58–80)
										Hospitalization	81 (49–93)
Goldberg et al. [53] (Israel)	Prospective cohort	20 Dec 2020–20 Mar 2021	General population (16+)	6,351,903 **	Not provided	5,682,928 ** (668,975)	BNT162b2	>7	*B.1.1.7*	Infection	93 (93–93)
										Hospitalization	94 (94–95)
										Death	94 (93–95)
										Severe disease	94 (94–95)
Guijarro et al. [54] (Spain) ^@^	Prospective cohort	21 Dec 2020–24 Apr 2021	HCWs (not provided)	2590	Not provided	2,116 (474)	BNT162b2	>0	NA	Infection	92 (83–96)
Hitchings et al. [55] (Brazil) ^@^	Test-negative case–control	19 Jan 2021–13 Apr 2021	HCWs (18+)	590	Not provided	50 (493)	CoronaVac	>14	*P.1*	Infection	38 (−46 to 74)
										Symptomatic	37 (−53 to 74)
						47 (493)	CoronaVac	0–13	*P.1*	Infection	50 (−2 to 76)
										Symptomatic	54 (−0.4 to 80)
Ismail et al. [56] (UK)	Case-coverage	8 Dec 2020–18 Apr 2021	Hospitalized COVID patients (80+)	2047	Not provided	27 (2010)	BNT162b2	>14	NA	Hospitalization	93 (89–95)
						10 (2010)	BNT162b2	7–13	NA	Hospitalization	88 (76–94)
Lopez Bernal et al. [57] (UK) ^@^	Test-negative case–control	26 Oct 2020–16 May 2021	General population (16+)	132,203	Not provided	15,798 (103,684)	BNT162b2	>14	**B.1.1.7**	Symptomatic	94 (92–95)
						15,871 (100,414)	BNT162b2	>14	**B.1.617.2**	Symptomatic	88 (85–90)
						8338 (103,684)	ChAdOx1	>14	**B.1.1.7**	Symptomatic	74.5 (68.4–79.4)
						8462 (100,414)	ChAdOx1	>14	**B.1.617.2**	Symptomatic	67 (61–72)
Lopez Bernal et al. [58] (UK)	Prospective cohort	8 Dec 2020–6 Apr 2021	Older adults (70+)	38,235	Not provided	191 (38,044)	BNT162b2	>7	NA	Death	69 (31–86)
Lumley et al. [59] (UK) ^@^	Prospective cohort	23 Apr 2020–28 Feb 2021	HCWs (not provided)	3542	39 (median)	1456 (2086)	BNT162b2, ChAdOx1	>14	**B.1.1.7**	Infection	90 (62–98)
										Symptomatic	100
Moustsen-Helms et al. [60] (Denmark)	Retrospective cohort	27 Dec 2020–18 Feb 2021	LTCF residents (not provided)	35,435	84 (median)	33,567 (1868)	BNT162b2	>7	NA	Infection	64 (14–84)
			HCWs (not provided)	320,013	47 (median)	80,839 (239,174)	BNT162b2	>7	NA	Infection	90 (82–95)
Public Health England [61] (UK)	Case-coverage	8 Dec 2020–12 Feb 2021	Older adults (>80)	8971	Not provided	62 (8909)	BNT162b2	>7	NA	Symptomatic	88 * (84–90)
Regev-Yochay et al. [62] (Israel) ^@^	Retrospective cohort	19 Dec 2020–14 Mar 2021	HCWs (18+)	8877	Not provided	7324 (1553)	BNT162b2	>11	NA	Infection	88 (83–92)
										Asymptomatic	65 * (45–79)
										Symptomatic	90 (84–94)
Shah et al. [63] (UK) ^@^	Retrospective cohort	8 Dec 2020–3 Mar 2021	HCWs (18–65)	144,525	44 (mean)	36,227 (30,268)	BNT162b2, ChAdOx1 (1%)	>14	NA	Infection	92 (83–96)
Shrestha et al. [64] (US)	Retrospective cohort	16 Dec 2020–15 May 2021	HCWs	46,866	Vaccinated 44 (mean); unvaccinated 40 (mean)	28,223 (18,643)	BNT162b2, mRNA-1273	>14	NA	Infection	97 (94–99)
Stowe et al. [65] (UK)	Test-negative case–control	12 Apr 2021–4 Jun 2021	Symptomatic cases (not provided)	14,019 **	Not provided	Not provided	BNT162b2	>0	**B.1.1.7**	Hospitalization	95 (78–99)
								>0	**B.1.617.2**	Hospitalization	96 (86–99)
							ChAdOx1	>0	**B.1.1.7**	Hospitalization	86 (53–96)
								>0	**B.1.617.2**	Hospitalization	92 (75–97)
Young-Xu et al. [66] (US) ^@^	Test-negative case–control	14 Dec 2020–7 Mar 2021	Veterans (VHA patients) (18+)	70,661	Not provided	5031 (65,630)	BNT162b2, mRNA-1273	>7	NA	Infection	94 (92–95)
										Symptomatic	91 (87–93)
										Hospitalization	89 (81–93)
										Death	99 (87–100)

CI = confidence interval; HCW = health care worker; HS = health system; IBD = inflammatory bowel disease; ICU = intensive care unit; LTCF = long-term care facility; NA = not applicable; SNF = skilled nursing facility; UK = United Kingdom; US = United States; VHA = Veterans Health Administration; VOC = variant of concern. ^++^ Article not identified by PubMed search criteria, identified by Pfizer; ** >1 dose; * Crude VE; ^@^ Preprint published prior to submission of this manuscript.

**Table 2:** Characteristics of abstracted articles presenting VE of COVID-19 vaccines against SARS-CoV-2 infection and other relevant outcomes, 1 January–30 June 2021.

Characteristic	Published, *n* (%)	Preprint, *n* (%)	Total, *N* (%)
Total	28 (100)	22 (100)	50 (100)
**Country**			
Brazil	0	2 (9.1)	2 (4.0)
Canada	0	1 (4.5)	1 (2.0)
Denmark	0	2 (9.1)	2 (4.0)
India	1 (3.6)	0	1 (2.0)
Israel	5 (17.9)	3 (13.6)	8 (16.0)
Italy	4 (14.3)	0	4 (8.0)
Spain	3 (10.7)	2 (9.1)	5 (10.0)
Sweden	0	1 (4.5)	1 (2.0)
Qatar	2 (7.1)	0	2 (4.0)
United Kingdom	4 (14.2)	7 (31.8)	11 (22.0)
United States	9 (32.1)	4 (18.2)	13 (26.0)
**Study population ^a^**			
General population (aged ≥16 years)	12 (42.9)	9 (40.9)	21 (42.0)
Older adults (aged ≥65 years) ^b^	4 (14.2)	7 (31.8)	11 (22.0)
Adult frontline workers	12 (42.9)	10 (45.5)	22 (44.0)
Other ^c^	1 (3.6)	1 (4.5)	2 (4.0)
**Study design ^a^**			
Test-negative case–control	4 (14.3)	6 (27.3)	10 (20.0)
Prospective cohort	8 (28.6)	7 (31.8)	15 (30.0)
Retrospective cohort	12 (42.9)	6 (27.3)	18 (36)
Case-coverage	1 (3.6)	2 (9.1)	3 (6.0)
Other ^d^	4 (14.2)	1 (4.5)	8 (16.0)
**Vaccine ^a^**			
Ad26.COV2.S	0	1 (4.5)	1 (2.0)
BNT162b2	21 (75.0)	15 (68.2)	36 (72.0)
mRNA-1273	4 (14.3)	2 (9.1)	6 (12.0)
ChAdOx1	2 (7.1)	2 (9.1)	4 (8.0)
CoronaVac	0	2 (9.1)	2 (4.0)
BNT162b2 and mRNA-1273	7 (25.0)	4 (18.2)	11 (22.0)
BNT162b2 and ChAdOx1	0	2 (9.1)	2 (4.0)
ChAdOx1 and Covaxin	1 (3.6)	0	1 (2.0)
BNT162b2, mRNA-1273, and ChAdOx1	1 (3.6)	0	1 (2.0)
Days after full vaccine dose ^a^			
≥7 days ^e^	16 (57.1)	12 (54.5)	28 (56.0)
≥14 days ^f^	12 (42.9)	10 (45.5)	22 (44.0)
Other ^g^	2 (7.1)	4 (18.2)	6 (12.0)
**Identified variants ^a^**			
B.1.1.7 (Alpha)	6 (21.4)	4 (18.2)	10 (20.0)
B.1.351 (Beta)	1 (3.6)	0	1 (2.0)
R.1	1 (3.6)	0	1 (2.0)
B.1.617	1 (3.6)	0	1 (2.0)
B.1.617.2 (Delta)	0	2 (9.1)	2 (4.0)
Multiple variants ^h^	4 (14.3)	3 (13.6)	7 (14.0)
**Circulating variants ^a^**			
B.1.1.7 (Alpha)	4 (14.3)	1 (4.5)	5 (10.0)
P.1 (Gamma)	0	1 (4.5)	1 (2.0)
B.1.1.7, B.1.351 (Alpha, Beta)	1 (3.6)	0	1 (2.0)
B.1.1.7, B.1.351, P.1 (Alpha, Beta, Gamma)	0	1 (4.5)	1 (2.0)
B.1.1.7, B.1.617.2 (Alpha, Delta)	0	1 (4.5)	1 (2.0)
Variant not specified	14 (50)	13 (59.1) ^i^	27 (54.0)
**Disease presentation ^a^**			
Asymptomatic	7 (28.0)	2 (9.1)	9 (18.0)
Symptomatic	13 (46.4)	9 (40.9)	22 (44.0)
Infection	22 (78.6)	15 (68.2)	37 (74.0)
**Disease severity ^a^**			
Hospitalization	8 (28.6)	8 (36.4)	16 (32.0)
ICU admission or severe disease	6 (21.4)	4 (18.2)	10 (20.0)
Death	5 (17.9)	6 (27.3)	11 (22.0)

^a^ Individual studies sometimes included more than one of the listed categories; categories will not sum to total N. ^b^ Includes one study that reported VE among persons aged >60 years. ^c^ Other study populations in published articles include airline passengers (*n* = 1), hospital patients (*n* = 1), and veterans with IBD/immunosuppression (*n* = 1). Other study populations in preprint articles include LTCF residents, older adults, HCWs, and severe risk individuals (*n* = 1); severe risk individuals (*n* = 1); hospitalized COVID patients (*n* = 1); and symptomatic cases (*n* =1). ^d^ Other study designs among published articles include cross-sectional (*n* = 1), outbreak investigation (*n* = 1), surveillance study (*n* = 1), and test-negative cohort (*n* =1). Other preprint study design was comparative effectiveness (*n* = 1). ^e^ Includes studies that calculated VE at 7–13 days, 7–28 days, >8 days, >11 days, and ≥7 days (BNT162b2) or >14 days (mRNA-1273). ^f^ Includes studies that calculated VE at >15 days and ≥15 days. ^g^ Includes studies that calculated VE at ≥0 days, >1 day, 0–13 days. ^h^ Study provided VE estimate for multiple variants together (did not stratify VE by variant). Includes published studies that provided a VE for B.1.1.7, B.1.351, B.1.617, and “wildtype strains” (*n* = 1); B.1.1.7, B1.177, P.1, and B.1.351 (*n* = 1); B.1.1.7 and B1.525 (*n* = 1); and B.1.1.7, B.1.427, B.1.429, and P.2 (*n* =1). Includes preprint studies that provided a VE for B.1.1.7, B.1.427, and B.1.429 (*n* = 1); B.1.351 and P.1 (*n* = 1); and B.1.1.7, P.1, and other VOC (*n* = 1). ^i^ Includes one article that provides VE for “earlier variants.”

## Supplementary Materials

The following are available online at https://www.mdpi.com/article/10.3390/vaccines10030393/s1, Supplementary Methods, Table S1: Study population classification scheme.
