# Supplementary material for: COVID-19 Vaccine Effectiveness: A Review of the First 6 Months of COVID-19 Vaccine Availability (1 January–30 June 2021)
Source: Vaccines (Basel). 2022 Mar 3;10(3):393. doi: 10.3390/vaccines10030393 (PMC8951318; doi:10.3390/vaccines10030393)
Supplement: Supplementary file 1 [file vaccines-10-00393-s001.zip › Supplementary Methods.pdf]

vaccines

MDPI

Supplementary Methods

A systematic literature search was conducted in PubMed to identify published articles describing observational studies written in English, published between January 1 and June 30, 2021, that assessed SARS-CoV-2 vaccine effectiveness against COVID-19–related outcomes (e.g., infection, hospitalization, severe disease, ICU admission, death) among fully vaccinated persons in real-world settings. The following keywords were used to search the PubMed database: (COVID OR "COVID19" OR "COVID-19" OR "Coronavirus Disease 2019" OR "SARS-CoV-2" OR "Severe Acute Respiratory Syndrome Coronavirus 2" OR "sarscov2" OR "Pfizer-BioNTech mRNA COVID-19 vaccine BNT162b2" OR "Pfizer-BioNTech" OR Pfizer OR "mRNA BNT162b2" OR "BNT162b2" OR Comirnaty OR "Moderna mRNA1273" OR "Moderna mRNA-1273" OR "mRNA1273" OR "mRNA-1273" OR Moderna OR "mRNA vaccine" OR "mRNA vaccines" OR "ChAdOx1 nCoV-19 adenoviral Astrazeneca" OR "ChAdOx1" OR AstraZeneca OR "AZD1222" OR Vaxzevria OR Covishield OR Janssen OR "Johnson & Johnson" OR "Johnson and Johnson" OR "Ad26.COV2.S" OR "JNJ-78436735" OR "Sinopharm vaccine" OR "Sinovac-CoronaVac" OR CoronaVac) AND (vaccin* OR immunis* OR immuniz*) AND (effectiveness OR vaccin* effectiveness OR vaccin* efficacy).

The PubMed database was searched according to the following search terms: (COVID[ti] OR "COVID19"[ti] OR "COVID-19"[ti] OR "Coronavirus Disease 2019"[ti] OR "SARS-CoV-2"[ti] OR "Severe Acute Respiratory Syndrome Coronavirus 2"[ti] OR "sarscov2"[ti] OR COVID[ot] OR "COVID19"[ot] OR "COVID-19"[ot] OR "Coronavirus Disease 2019"[ot] OR "SARS-CoV-2"[ot] OR "Severe Acute Respiratory Syndrome Coronavirus 2"[ot] OR "sarscov2"[ot] OR "Pfizer-BioNTech mRNA COVID-19 vaccine BNT162b2"[tiab] OR "Pfizer-BioNTech"[tiab] OR Pfizer[ti] OR Pfizer[ot] OR "mRNA BNT162b2"[tiab] OR "BNT162b2"[tiab] OR Comirnaty[tiab] OR "Moderna mRNA1273"[tiab] OR "Moderna mRNA-1273"[tiab] OR "mRNA1273"[tiab] OR "mRNA-1273"[tiab] OR Moderna[ti] OR Moderna[ot] OR "mRNA vaccine"[tiab] OR "mRNA vaccines"[tiab] OR "ChAdOx1 nCoV-19 adenoviral Astrazeneca"[tiab] OR "ChAdOx1"[tiab] OR AstraZeneca[ti] OR AstraZeneca[ot] OR "AZD1222"[tiab] OR Vaxzevria[tiab] OR Covishield[tiab] OR Janssen[ti] OR Janssen[ot] OR "Johnson & Johnson"[ti] OR "Johnson & Johnson"[ot] OR "Johnson and Johnson"[ti] OR "Johnson and Johnson"[ot] OR "Ad26.COV2.S"[tiab] OR "JNJ-78436735"[tiab] OR "Sinopharm vaccine"[tiab] OR "Sinovac-CoronaVac"[tiab] OR CoronaVac[tiab] OR "COVID-19"[Mesh] OR "SARS-CoV-2"[Mesh] OR "COVID-19 Vaccines"[Mesh]) AND (vaccin*[ti] OR immunis*[ti] OR immuniz*[ti] OR vaccin*[ot] OR immunis*[ot] OR immuniz*[ot] OR "COVID-19 Vaccines"[Mesh]) AND (effectiveness[tiab] OR effectiveness[ot] OR "vaccine effectiveness"[tiab] OR "vaccines effectiveness"[tiab] OR "vaccine's effectiveness"[tiab] OR "vaccination effectiveness"[tiab] OR "vaccine efficacy"[tiab] OR "vaccines efficacy"[tiab] OR "vaccine's efficacy"[tiab] OR "vaccination efficacy"[tiab]) AND ("2021/01/01"[Date - Publication] : "2021/06/30"[Date - Publication]) Filters: English
