# Supplementary material for: COVID-19 Vaccine Effectiveness: A Review of the First 6 Months of COVID-19 Vaccine Availability (1 January–30 June 2021)
Source: Vaccines (Basel). 2022 Mar 3;10(3):393. doi: 10.3390/vaccines10030393 (PMC8951318; doi:10.3390/vaccines10030393)
Supplement: Supplementary file 1 [file vaccines-10-00393-s001.zip › Supplementary Table S1.pdf]

vaccines

MDPI

Table S1. Study population classification scheme.

Population group[a]	Study population[b]
General population	Airline passengers; general population; health system (HS) members; HS members (close contacts); veterans (VHA patients); symptomatic cases
Older adults	Skilled nursing facility (SNF) residents; older adults; long-term care facility (LTCF) residents; hospital patients (aged ≥65 years); older adults requiring help; hospitalized COVID patients (aged ≥80 years)
Adult frontline workers	Healthcare workers (HCWs); SNF HCWs; HCWs, first responders, and essential workers; LTCF staff; vaccinated HCW and general population[c]
Other	Combined category of LTCF residents, older adults, HCWs, severe risk individuals; veterans with IBD/immunosuppressed; severe risk individuals

VHA = Veterans Health Administration
[a]Used in Table 1, Figures 2-4, and written results.
[b]Used in Table 2.
[c]This study compared vaccinated HCWs to unvaccinated persons in the general population.
